# Evaluation of the interhospital patient transfer after implementation of a regionalized trauma care system (TraumaNetzwerk DGU^®^) in Germany

**DOI:** 10.3389/fmed.2023.1298562

**Published:** 2023-11-15

**Authors:** C. Spering, D. Bieler, S. Ruchholtz, B. Bouillon, R. Hartensuer, W. Lehmann, R. Lefering, H. Düsing, for Committee on Emergency Medicine, Intensive Care and Trauma Management (Sektion NIS) of the German Trauma Society (DGU)

**Affiliations:** ^1^Department of Trauma Surgery, Orthopedics and Plastic Surgery, Göttingen University Medical Center, Göttingen, Germany; ^2^Department of Orthopaedics and Trauma Surgery, Heinrich Heine University Medical School, Düsseldorf, Germany; ^3^Department of Orthopaedics and Trauma Surgery, Reconstructive Surgery, Hand Surgery, Plastic Surgery and Burn Medicine, German Armed Forces Central Hospital, Koblenz, Germany; ^4^Center for Orthopedics and Trauma Surgery, University Hospital Giessen and Marburg, Marburg, Germany; ^5^Department of Trauma Surgery, Orthopedics and Sports Traumatology, University of Witten/Herdecke, Cologne, Germany; ^6^Center for Orthopaedics, Trauma Surgery, Hand Surgery and Sports Medicine, Surgical Clinic II, Klinikum Aschaffenburg-Alzenau, Aschaffenburg, Germany; ^7^Institute for Research in Operative Medicine (IFOM), University of Witten/Herdecke, Cologne, Germany; ^8^Department of Trauma, Hand and Reconstructive Surgery, University Hospital Münster, Münster, Germany

**Keywords:** trauma, trauma care, interhospital transfer, trauma care system, TraumaRegister DGU^®^, TraumaNetwork DGU^®^, polytrauma management

## Abstract

**Purpose:**

The aim of the study was to evaluate how many patients are being transferred between trauma centers and and their characteristics in the 2006 initiated TraumaNetzwerk DGU^®^ (TNW). We further investigated the time point of transfer and differences in outcome, compared to patients not being transferred. We wanted to know how trauma centers judged the performance of the TNW in transfer.

**Method:**

(1) We analyzed the data of the TraumaRegister DGU^®^ (TR-DGU) from 2014–2018. Included were patients that were treated in German trauma centers, maximum AIS (MAIS) >2 and MAIS 2 only in case of admission on ICU or death of the patient. Patients being transferred were compared to patients who were not. Characteristics were compared, and a logistic regression analysis performed to identify predictive factors. (2) We performed a survey in the TNW focussing on frequency, timing and communication between hospitals and improvement through TNW.

**Results:**

Study I analyzed 143,195 patients from the TR-DGU. Their mean ISS was 17.8 points (SD 11.5). 56.4% were admitted primarily to a Level-I, 32.2% to a Level-II and 11.4% to a Level-III Trauma Center. 10,450 patients (7.9%) were transferred. 3,667 patients (22.7%) of the admitted patients of Level-III Center and 5,610 (12.6%) of Level-II Center were transferred, these patients showed a higher ISS (Level-III: 18.1 vs. 12.9; Level-II: 20.1 vs. 15.8) with more often a severe brain injury (AIS 3+) (Level-III: 43.6% vs. 13.1%; Level-II: 53.2% vs. 23.8%). Regression analysis showed ISS 25+ and severe brain injury AIS 3+ are predictive factors for patients needing a rapid transfer. Study II: 215 complete questionnaires (34%) of the 632 trauma centers. Transfers were executed within 2 h after the accident (Level-III: 55.3%; Level-II: 25.0%) and between 2–6 h (Level-III: 39.5%; Level-II: 51.3%). Most trauma centers judged that implementation of TNW improved trauma care significantly (Level III: 65.0%; Level-II: 61.4%, Level-I: 56.7%).

**Conclusion:**

The implementation of TNW has improved the communication and quality of comprehensive trauma care of severely injured patients within Germany. Transfer is mostly organized efficient. Predictors such as higher level of head injury reveal that preclinical algorithm present a potential of further improvement.

## Introduction

Major trauma remains the main cause of disability and death worldwide, especially among young and economically active adults (1). Regionalization of trauma care within a network of hospitals was initiated in Germany in 2006 (2). This initiative was started due to an increasing number of hospitals quitting from trauma care of severely injured patients at that time. Evaluation of the quality of care showed significant regional differences in mortality rate (2). Emergency services complained about difficulties to find hospitals ready to admit trauma patients. Reasons for this were inadequate reimbursement of hospitals, a reduction of staff in the emergency rooms and a shift towards economically more interesting elective patients (2, 3). The Implementation of TraumaNetzwerk DGU^®^ (TNW) was completed in 2015. Today Germany is covered by 50 regionalized trauma care networks. Each network consists of designated trauma centers Level-I-III that support each other according to defined guidelines and regulations (4). Experience from such trauma systems and the effort to optimize trauma care reveal improvement of patient’s outcome over the last 20 years in Germany (2), the Netherlands, Norway (5), the United Kingdom (5, 6) and the United States (6, 7). It appears that a structured and nationally organized trauma care from the scene of accident to rehabilitation has a higher impact on outcome than any single medical intervention (6). Elements of this systematic approach are the Level III national interdisciplinary guideline (S3-LL) (8), the nationwide implementation of Advanced Trauma Life Support (ATLS) in Germany since 2003 and the continuous feedback from the German Trauma Register (TraumaRegister DGU^®^, TR-DGU) since 1993 to ensure quality of care within the regionalized networks (3, 8).

The aim of a nationwide structured trauma system such as the TNW is to assure comprehensive quality of trauma care nationwide, measured by survival and quality of life (2–4, 8, 9). It therefore attempts to strengthen the quality of care also in rural areas and smaller hospitals through cooperation between trauma centers of different levels of trauma care. It is supposed to create a network that provides a foundation to initially admit trauma patients to any participating trauma center, stabilize them according to defined trauma care standards and to organize rapid secondary interhospital transfer of severely injured patients if necessary (2–6, 10, 11).

Providing trauma care in designated trauma centers can save lives and prevent long-term disability (6), thus direct transportation of severely injured patients to designated centers, while bypassing closer non-specialized facilities, is considered beneficial. Few studies have analysed the relationship between mortality rate and primary or secondary transport to a Level-I Trauma Center (9, 11–14). These studies have been exclusively conducted in paramedic staffed prehospital emergency systems without physicians being involved on scene (13, 14). Hamada et al. (11) were most recently able to show in France that the direct vs. secondary transport of severely injured patients seem to not have an influence on their outcome in a physician-based prehospital trauma team. Elderly severely injured patients though seem to be at risk of a preclinical undertriage and favourably transported to the nearest trauma center regardless of its grade of speciality (15). In all these scenarios though, we do not know enough about these patients to estimate their outcome if they had been transported to a center with a higher level of trauma care.

Although multiple quality indicators have been identified as predictors for improved outcome of severely injured patients being treated in such national trauma systems, it has been impossible to prove the benefit for patients, who are in need of a rapid transfer in the early stage of trauma management. There are many factors associated with the decision to transfer trauma patients to a Level-I Trauma Center, influencing the final decision making process.

The purpose of this study was to evaluate how many patients are being transferred between trauma centers within the TNW and who they are. We further wanted to investigate when they were transferred and if their outcome differed compared to patients that were not transferred. Finally, we wanted to know how trauma centers judged the performance of the TNW, including transfer management, communication and aftercare of the patients.

## Methods

### The TraumaRegister DGU^®^

The TraumaRegister DGU^®^ (TR-DGU) of the German Trauma Society (Deutsche Gesellschaft für Unfallchirurgie, DGU) started 1993. The aim of this multi-centre database is the pseudonymised and standardized documentation of severely injured patients. Data are collected prospectively in four consecutive time phases from the site of the incident until discharge from hospital: (A) prehospital phase, (B) emergency/resuscitation room and initial surgery, (C) intensive care unit, and (D) discharge. Documentation includes detailed information on demographics, injury patterns, comorbidities, prehospital and in-hospital management, course on intensive care unit, relevant laboratory findings including transfusion data, and outcome. Included are patients who are admitted to hospital via the resuscitation room and subsequently receive intensive or intermediate care and patients who arrive at hospital with vital signs and die before admission to the intensive care unit.

The infrastructure for documentation, data management, and data analysis is provided by the Academy for Trauma Surgery (Akademie der Unfallchirurgie GmbH, AUC), which is affiliated with the German Trauma Society. Scientific leadership is provided by the Committee on Emergency Medicine, Intensive Care and Trauma Management (Sektion NIS) of the German Trauma Society. Participating hospitals submit their pseudonymised data to a central database via a web-based application. Scientific data analysis is approved according to a peer review procedure established by Sektion NIS.

The participating hospitals are primarily located in Germany (90%), but a growing number of hospitals in other countries contribute data as well (i.e., Austria, Belgium, Finland, Luxembourg, Slovenia, Switzerland, the Netherlands, and the United Arab Emirates). Currently, approximately 30,000 cases (basic group of patients) from more than 650 hospitals are entered into the database per year. Participation in TR-DGU is voluntary. For hospitals associated with the TNW, the entry of at least a basic data set is compulsory for reasons of quality assurance. Approximately 50% of all cases, however, are documented on the base of the standard dataset.

### Study I: analysis of the TraumaRegister DGU^®^

The first study included patients from the registry admitted to a German hospital between 2014 and 2018. Patients with a maximum AIS ≥ 3 (MAIS ≥3) and patients with a MAIS 2 who died or were treated on an intensive care unit were included. The evaluation was performed for Level I, II and III Trauma Centers. The control group consisted of primary admissions without transfer (within the first 48 h). An exact matching of patients transferred out from one hospital and then admitted to another hospital was not possible due to the lack of a uniform case identifier but the patients are either labeled as being transferred or primarily treated in the TraumaRegister DGU^®^. Furthermore, date and time of transfers were removed from the scientific dataset due to data protection reasons.

For descriptive statistics mean with SD was used for continuous measurements, and *n* with percent was used for categorical variables. In addition, a logistic regression analysis was performed in primary admitted patients to identify factors associated with an early transfer out (dependent variable) from Level-II and III Trauma Centers. Independent variables were young age (<16), injury severity (ISS <16 /16–24, 25+), intubation prehospital, unconsciousness (Glasgow Coma Scale GCS ≤ 8) and serious head injury (AIS 3+). Results are shown as odds ratios with 95% confidence intervals.

Statistics were performed with SPSS^®^ (Version 24, IBM Inc., Armonk, NY, United States).

This study is in accordance with the publication guideline of the TR-DGU and is registered under the TR-DGU project ID 2019–020.

### Study II: survey with questionnaire distributed to trauma centers

The second study was a survey with a questionnaire that was distributed to 632 Trauma Centers that take part in the TNW. The idea was to collect the trauma centers perception of daily life reality. We therefore developed a questionnaire that addressed relevant questions in the context of patient transfer between trauma centers. The following five topics were included in the questionnaire: (1) Frequency of patient transfer independently from injury severity, (2) Timing of patient transfer, (3) Communication around the patient transfer, (4) Management of the aftercare of severely injured transferred patients, and (5) General subjective perception of the impact of TNW.

The administrators of all German certified trauma centers (*n* = 632) where contacted via email, including an online link to participate in this survey and anonymously evaluated afterwards. If the questionnaire was not returned within 4 weeks a second memo was sent to the trauma centers. For descriptive statistics mean with SD was used and *n* with percent was used for categorical variables for every trauma center level.

## Results

### Study I: data from the TraumaRegister DGU^®^

143,195 patients met the inclusion criteria of whom 69.9% were male, the mean age was 52.1 years (SD 22.5) and the mean ISS was 17.8 points (SD 11.5). 132,086 patients were primary admitted to a trauma center, the other patients were transfers. Among the primary admitted patients, 121,636 patients (92.1%) were definitely treated at the hospital of initial admission and 10,450 (7.9%) were transferred to another trauma center. 54.1% were admitted primarily to a Level-I, 33.7% to a Level-II and 12.2% to a Level-III Trauma Center. The overall mortality rate of the study population was 9.8%.

#### Transfer depending on designated level of trauma care

**Figure 1:**
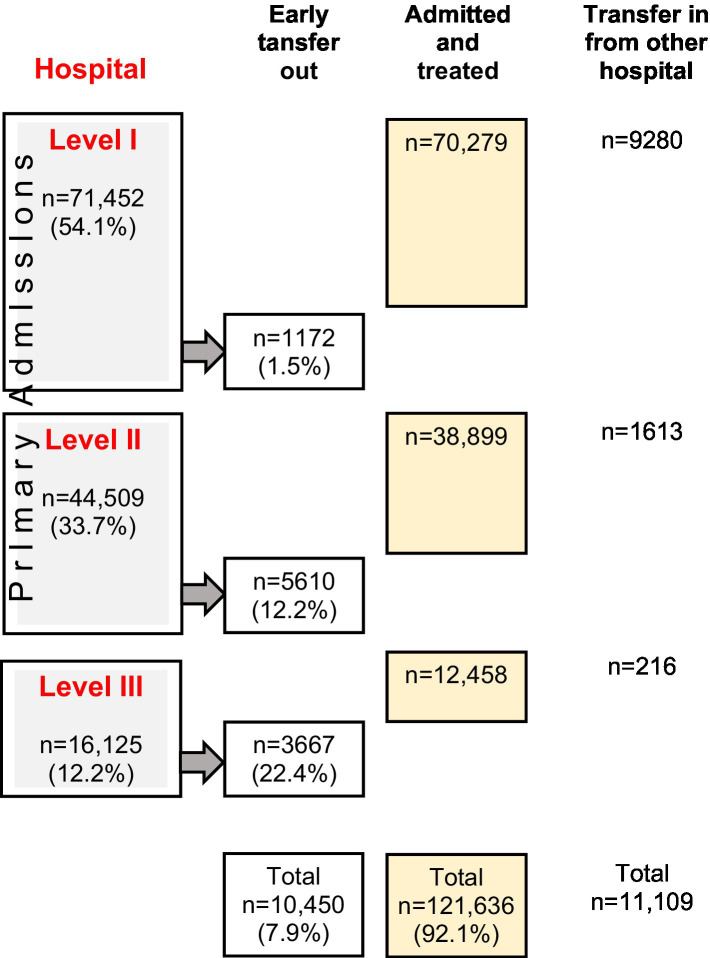
Transfer depending on designated level of trauma care, data from TR-DGU.

Most of the severely injured patients were treated primarily in the trauma center that they got initially admitted to (92.2%) (Table 1; Figure 1). Of the 11,191 patients (7.8%), who were transferred to another trauma center, 3,667 patients (22.7%) were transferred out from Level-III and 5,610 patients (12.6%) were transferred out from Level-II trauma centers. Among the 11,109 cases documented as secondary admissions, most cases were received by Level-I Trauma Centers (*n* = 9,280; 83.5%) while only 1,613 patients (14.5%) were received by Level-II Trauma Centers (Table 1).

**Table 1:** Comparison of demographic and injury data of study patients primary admitted to a Level-II or Level-III Trauma Center who were either treated in that hospital, or transferred out early to another hospital (<48 h) and of patients transferred in to Level-I and -II Trauma Centers, compared to those admitted and treated in these trauma centers.

Level of care	Level III		Level II			Level I	
	Treated	Transfer out	Treated	Transfer out	Transfer in	Treated	Transfer in
	*n* = 12,458	*n* = 3,667	*n* = 38,899	*n* = 1,613	*n* = 1,613	*n* = 70,279	*n* = 9,280
Age (years)	55 (23)	53 (22)	53 (22)	50 (22)	59 (22)	50 (22)	56 (23)
Children (<16 years)	2.00%	3.70%	2.60%	5.20%	2.50%	4.30%	3.70%
Male sex (%)	65.70%	69.80%	68.00%	71.90%	67.60%	71.00%	70.00%
ISS (mean)	12.9 (8.5)	18.1 (9.3)	15.8 (10.2)	20.1 (10.3)	19.9 (10.2)	19.1 (12.4)	21.2 (11.3)
AIS Head 3+ (%)	13.90%	30.40%	23.80%	31.20%	58.20%	38.60%	57.40%
AIS Thorax 3+ (%)	35.20%	30.40%	37.60%	31.20%	28.50%	38.30%	32.00%
AIS Abdomen 3+ (%)	6.70%	10.70%	8.50%	8.90%	8.70%	9.60%	10.20%
GCS < 9 (%)	4.30%	9.70%	9.20%	14.70%	–	20.60%	–
Blood transfusion (%)	3.10%	4.80%	4.40%	4.90%	4.80%	8.60%	7.60%
Emergency surgery (%)	14.50%	10.60%	21.70%	10.90%	23.60%	27.40%	25.90%
Whole-body CT (%)	61.20%	65.70%	76.10%	70.30%	26.00%	83.80%	34.60%
Admission at night (%)	34.50%	37.80%	36.60%	41.00%	43.10%	38.10%	48.00%
Admitted to ICU	85.70%	44.90%	90.00%	50.70%	89.70%	92.70%	96.10%
Estimated risk of death (based on RISC II) (%)	5.70%	8.60%	7.90%	9.80%	–	11.60%	–
Hospital mortality (%)	5.70%	–	8.60%	–	13.30%	12.30%	12.90%

ISS, injury severity score; AIS, abbreviated injury scale; GCS, Glasgow coma scale; CT, computed tomography; ICU, intensive care unit.

#### Patients at level-III trauma centers

The mean ISS in the group of transferred patients was 18.1 points compared to 12.9 points in patients that had not been transferred out. The transferred patients showed twice as often a low GCS (3–8) compared to the non-transferred patients and suffered from a severe head Injury (AIS 3+) more often (43.6% vs. 13.9%). Emergency surgery had been performed less often in transferred patients (10.6% vs. 14.5%). The time of admission as well as other severe injuries (AIS 3+) such as thoracic trauma and abdominal injuries did not show a difference in the two groups (Table 1). Level-III Trauma Centers transferred out every third child (35%), unknown whether to a Level-I Trauma Center or to a designated pediatric trauma center.

#### Patients at level-II trauma centers

Patients admitted to Level-II Trauma Centers and rapidly transferred out showed more often a relevant (AIS 3+) brain injury (53.2% vs. 23.8%) compared to patients treated in the Level-II Trauma Centers. They were more often unconscious (GCS 3–8, 14.7% vs. 9.2%) and their mean ISS was higher (20.1 points vs. 15.8 points). Emergency surgery was performed half as often in the transferred group (10.9% vs. 21.3%). No differences were seen in thoracic injuries, abdominal injuries and blood transfusions (Table 1).

#### Patients at level-I trauma centers

Patients being transferred to Level-I Trauma Centers within 48 h after the initial trauma were older (56 vs. 50 years) and showed a higher mean ISS (21.0 vs. 19.1 points) compared to patients primarily admitted to a Level-I Trauma Center. A severe traumatic brain injury (AIS 3+) was seen more often compared to the primarily admitted patient group (58% vs. 39%). The mean/median duration of stay on the ICU was longer in transferred patients (9.2/4 vs. 6.8/2 days). There was no difference in abdominal and thoracic injuries. Half of the transferred patients were admitted to the Level-I Trauma Center during night time (50%). There were no significant differences in mortality between transferred and primarily treated patients at Level-I Trauma Centers (13.1% vs. 12.3%) (Table 1).

#### Time of transfer

**Figure 2:**
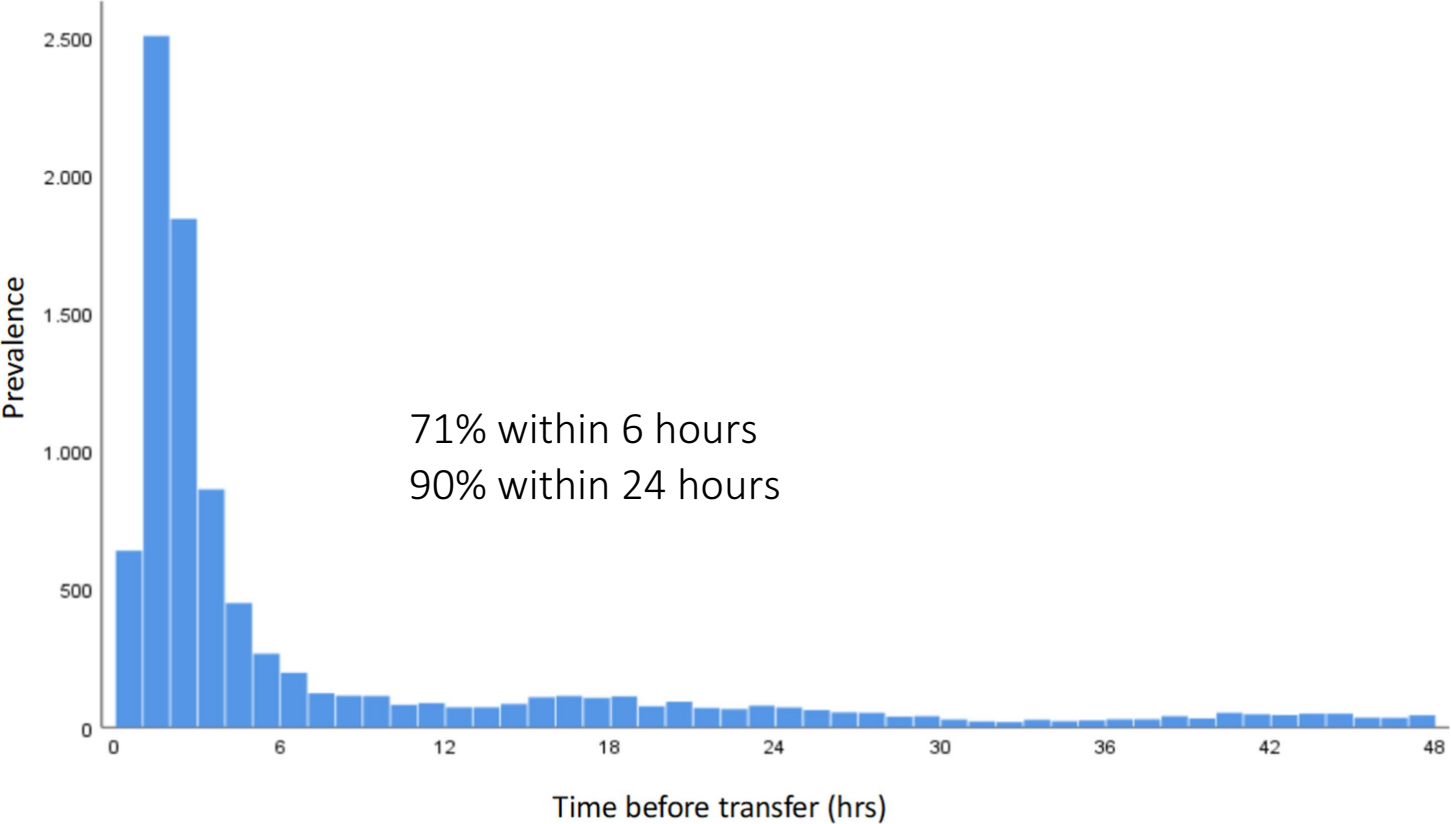
Time until transfer (hours) in all patients transferred out within 48 h after admission. The average and median time until transfer was 7.9 and 2.8 h, respectively.

Patients who were transferred within the TNW were mostly rapidly transferred. 90% of transfers occured within the first 24 h, 70% were transferred within 6 h after initial admission to a trauma center. The median duration of stay in the first trauma center before being transferred was 2.8 h (Figure 2).

#### Patients being transferred to a level-I trauma center

At time of admission at a Level-I Trauma Center, 40% of the transferred patients had already been intubated, only 7% were in shock and coagulopathy was observed in 15%. More than half of the patients (57%) had received some type of CT-scan prior to the transfer. 35% were taken to get a whole-body CT after having been transferred and admitted to a Level-I Trauma Center. Most transferred patients (64%) got directly admitted to intensive care unit (ICU) after management in the Trauma Resuscitation Unit (TRU). 28% of the transferred patients were taken directly from the TRU to the operating theatre (Table 1).

#### Predictors for transfer

An additionally performed logistic regression analysis (*n* = 52,130 from Level II and III) with the dependent variable *early transfer out* (within 48 h after admission) showed that a severe traumatic brain injury (AIS 3+) with an OR of 2.83 [CI 2.67–3.01] is a predictor for early transfer and so is severe trauma (ISS 25+) with an OR = 2.52 [CI 2.35–2.71]. Age ≤ 16 is also seen as a predictor for an early transfer with an OR of 1.59 [CI 1.39–1.83]. Prehospital intubation was no predictor for early transfer, on the contrary, these patients were less transferred with an OR = 0.62 [0.54–0.67] (Table 2).

**Table 2:** Logistic regression analysis in patients primary admitted to Level II and Level III trauma centers, with early transfer out as dependent variable.

	Odds ratio (OR)	95% CI for OR	*p* value
Head injury AIS 3+	2.83	2.67–3.01	<0.001
ISS 25+ (ref: <16)	2.52	2.35–2.71	<0.001
ISS 16–24 (ref: <16)	1.84	1.73–1.96	<0.001
Young age (<16 years)	1.59	1.39–1.83	<0.001
Shock on admission	1.07	0.96–1.20	0.223
Admission during the night	1.06	1.00–1.11	0.041
Need for blood transfusion	1.00	0.89–1.13	0.985
GCS 3–8	0.99	0.89–1.10	0.876
Admission during the weekend	0.88	0.83–0.92	<0.001
Old age (65+ years)	0.67	0.63–0.71	<0.001
Intubation prehospital	0.60	0.54–0.67	<0.001
Constant	0.10		

52,130 patients have had complete data in all predictor variables. Predictors were ordered according to decreasing odds ratio (OR). An OR > 1.0 favours an early transfer to another hospital while an OR < 1 did not. Nagelkerke’s r^2^ was 0.11.

### Study II: data acquisition through a questionnaire within the TNW

Out of 632 contacted trauma centers within Germany, 215 (34%) replied and handed in a completed questionnaire. 25.6% of the replies came from Level-I, 35.8% from Level-II and 38.6% from Level-III Trauma Centers.

#### Reason for transfer

Out of the replies from the questionnaire the main reason for transfer (83.6%) were medical specialty (i.e., neurosurgery, cardio-thoracic-surgery, burns) for Level III and II Trauma Centers followed by transfer due to overall higher level of trauma care and capacity problems in ICU or OR. Capacity problems were also a top 4 reason for transfer out of Level I Trauma Centers as well as repatriation, which was as well a frequent reason for receiving transferred patients in Level-III Trauma Centers (Table 3).

**Table 3:** “What are the 4 main reasons why your Trauma Center has received or transferred patients?”; data from the questionnaire in %.

	Level III		Level II		Level I	
	Received	Transferred	Received	Transferred	Received	Transferred
Higher level of designated Trauma Care within TNW	/	76.7	76.7	52.6	51.4	/
Insufficient ICU-capacity	27.3	26.0	26.0	12.8	28.6	20.3
Insufficient OR-capacity	20.5	20.5	9.6	10.3	25.7	8.5
Medical speciality (Neurosurgery, Cardio-Thoracic-Surgery, Burn-Unit etc.)	9.1	83.6	83.6	76.9	31.4	50.2
Complication	4.5	9.6	9.6	7.7	7.1	8.5
Repatriation	52.3	8.2	8.2	16.7	37.1	32.2
Other	3.2	1.4	1.4	5.1	11.4	22

#### Frequency and timing of patient transfer independently from injury severity

**Figure 3:**
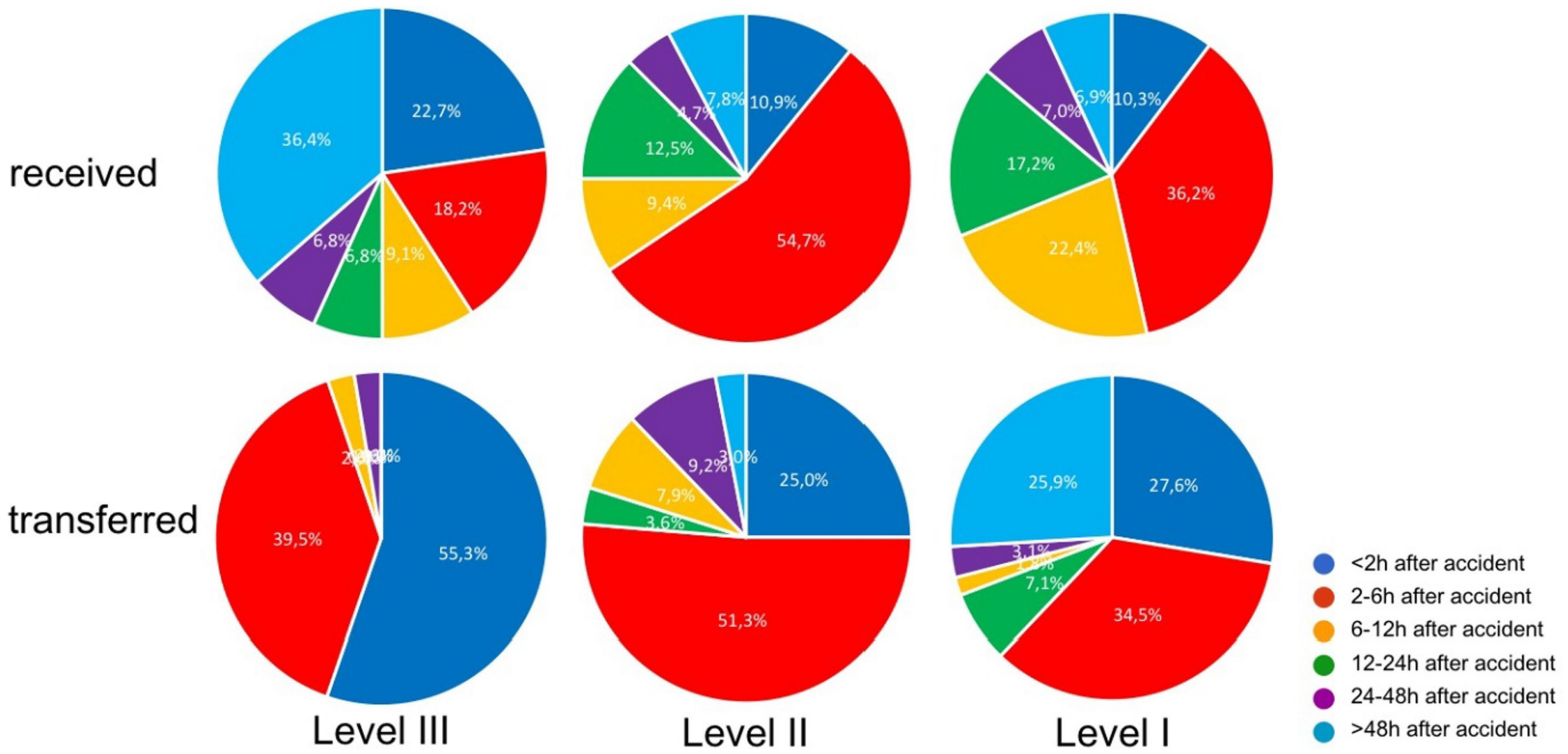
Transfer-timing in comparison of received versus transferred patients in dependence of the level of trauma care (Level-I, -II, and -III Trauma Center), in percent; data from the questionnaire.

**Figure 4:**
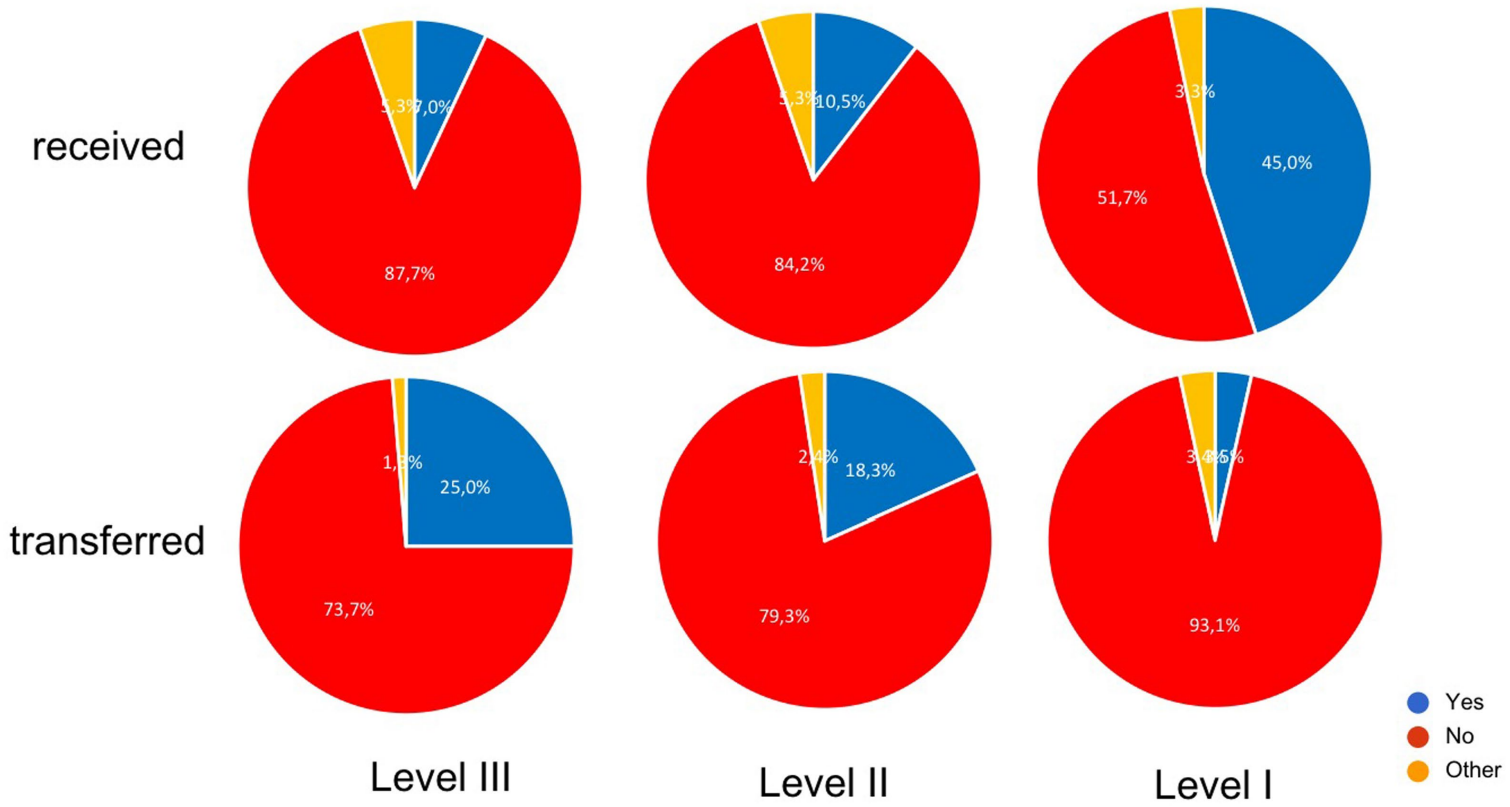
Evaluation of the questionnaire, if the trauma centers have been receiving more patients through a trauma-network-managed transfer. Level-III Trauma Centers have been transferring more patients in 25% and Level-I Trauma Center have been receiving more patients in 45% since the TNW had been established.

The data analysis of the questionnaire showed that the majority of patients were estimated being transferred within 2–6 h after accident (rapid/early transfer) (Figure 3). The Level-III Trauma Centers showed the highest rate of rapid transfer (94.8% < 6 h). If Level-III Trauma Centers had received patients to their hospital, they mostly received “late transferred” patients (50% after >24 h) (See Figure 4).

#### Communication around the patient transfer

Being one of the major criteria in assessing the quality and the process of patient transfer, communication before and after the transfer played a relevant role in the survey. The respondents rated the general communication between the trauma centers with “good”. The communication was mostly (92.2%–100%) via direct doctor to doctor call. Requested transfers from Level-III or Level-II to Level-I were described in 3.0%–8.4% as delayed due to capacity problems, this occasionally (9.2%) happened in the other direction (Level-I to Level-II/III) as well (See Table 4).

**Table 4:** Data transfer of diagnostics; data from the questionnaire in %.

	Level III		Level II		Level I	
	Received	Transferred	Received	Transferred	Received	Transferred
Transmitted prior to transfer via telecommunication (i.e., Tkmed^®^)	38.5	73.6	40.6	73.4	58.3	51.9
Together with patient via data medium (i.e., CD)	51.9	23.6	56.5	22.8	40.0	46.3
Only in written format	2.4	1.4	0.0	0.0	0.0	0.0
Other	7.2	1.4	2.9	3.8	1.7	1.8

Not only the communication prior to a patient transfer is of interest, but also afterwards, to ensure quality or optimize the process. While 75% of the receiving trauma centers responded that they sent out discharge letters to the transferring trauma centers regularly, only 55% of the transferring hospitals responded that they frequently received a discharge letter from the receiving trauma center. An immediate feedback on the quality of the transfer occurs in only 20% (Table 5).

**Table 5:** Communication after transfer; data from the questionnaire in %.

	Level III		Level II		Level I	
	Received	Transferred	Received	Transferred	Received	Transferred
Direct Feedback after transfer	73.6	30.7	75.7	41.5	58.3	51.8
Discharge letter of patient’s clinical course	60.8	60.8	58.4	53.7	78.0	53.6
Collective overall evaluation	13.5	13.5	18.1	16.0	12.1	13.0

#### Management of the aftercare of severely injured transferred patients

The respondents of the questionnaire stated that initially transferred patients got sent back after surgical and/or intensive care treatment in 10%–15% to the initial transferring trauma center. Regarding to the answers, the aftercare of former polytraumatized patients mostly takes place in Level-I Trauma Centers. 49% of them indicated, that they manage the aftercare of the initially transferred polytraumatized patients *regularly*, 42% seldomly. Independently of a transfer, 40% of the Level-I Trauma Centers indicate to manage the aftercare of polytraumatized patients at all. This applies to 30% of the Level-II and 20% of the Level-III Trauma Centers.

#### General subjective appraisal of the TNW

**Figure 5:**
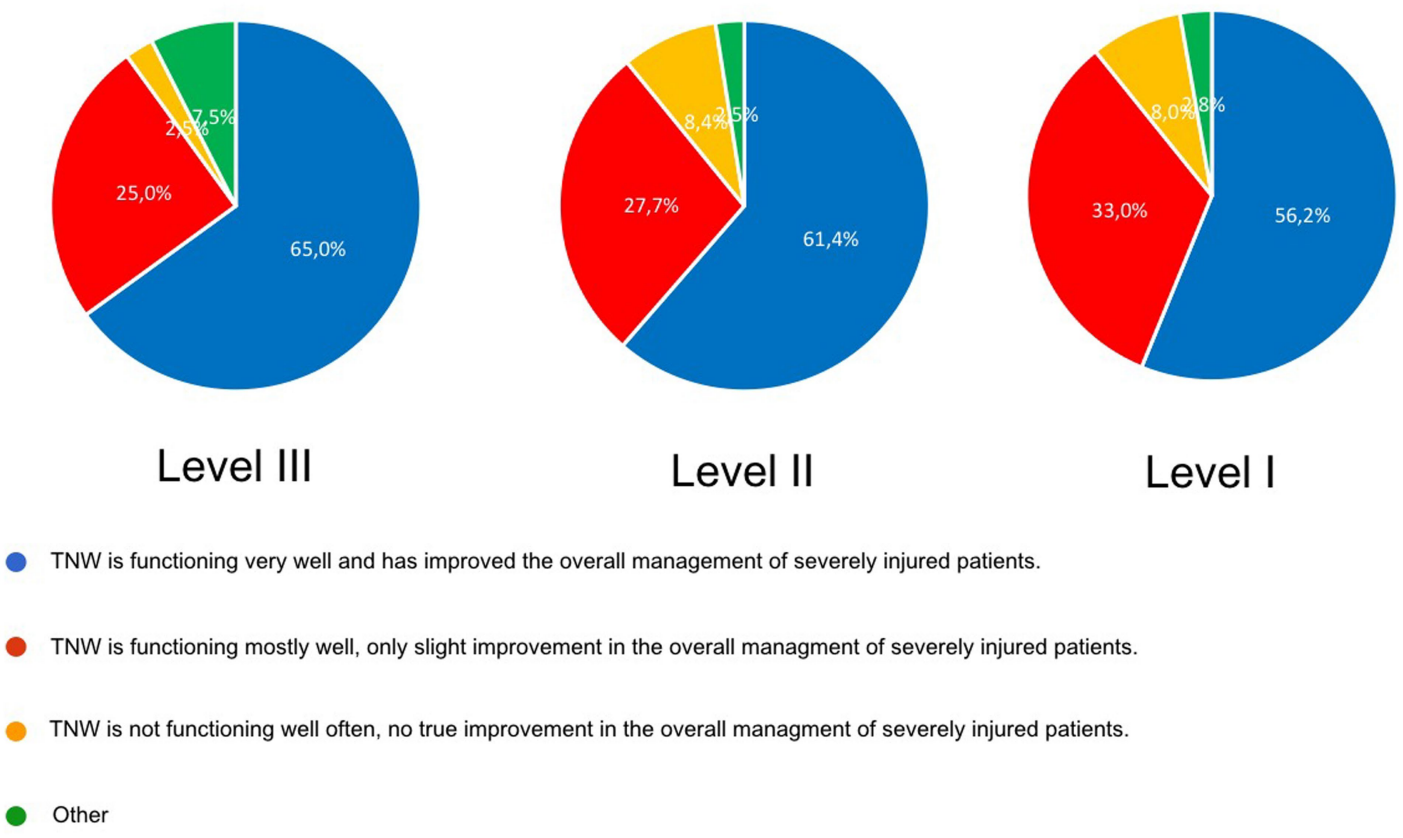
General evaluation of the improvement of patient transfer and management of severely injured patients since establishment of the TNW; data from the questionnaire.

The participating trauma centers were asked if they felt that overall TNW had improved the care of severely injured patients. Most participants judged that TNW was functioning mostly or very well with some improvement of the overall management Many trauma centers judged that implementation of TNW improved trauma care significantly (Figure 5).

## Discussion

The presented data allow some detailed insight to interhospital transfer of severely injured patients within the TNW in Germany.

The results show that Level-I and Level-II Trauma Centers carry the main load in receiving and finally treating severely injured patients of the TNW. 92.2% of these patients were treated up to their discharge in the trauma center they were primarily admitted to. The high percentage of patients treated in the hospital they were primaly admitted to seems to be an indicator for a good working prehospital triage system. Similar findings show Tiruneh et al. for transferd patients in Israel. In the evaluated population only 9.5% of the patients been transferred to another Trauma Center. Those patients were similar to our results more often under 16 years and had more often severe head injuries. Tiruneh et al. showed a greater risk of in-hospital mortality, we could not show this for our population taking the limitations (i.e., RISC Score) into account (16).

Schneppendahl et al. (17) have also analyzed data from the TR-DGU, at an earlier period of time (2002–2007) before initiation of the nationwide TNW and found 84.2% of patients to be treated at the hospital they were initially admitted to until discharge (17). Compared to that earlier study the number of initially correct admissions seems to rise (92.2% vs. 84.2%). This could be a result of successful networking within the TNW and an improved communication and training between prehospital emergency medical services and admitting trauma centers.

The fact that transferred patients were more severely injured might as well show successful networking if patients were stabilized in the lower level Trauma Center and transferd afterwards.

Joosse et al. (18) were able to show in their subgroup analysis for transferred patients with severe traumatic brain injuries in the Netherlands prolonged accident-to-surgery-times and a worse outcome. But there might be a bias due to only transferring patients who were stable enough for being transferred in the first place.

The short time between admission and transfer might also be an achievement of the TNW.

This is in concordance with the answers from the survey, where respondents stated, that the predominant reasons for a needed transfer are due to specific medical specialties and necessity of a higher level of trauma care.

Interesting enough, the survey also revealed a lack of resources being stated as a reason for early transfer of patients.

On one side one could argue that the responsibility of TNW could be to balance out limited resources and still secure a good quality of care. On the other side it should be discussed that any transfer that has not been initiated due to medical reasons but due to a lack of resources, is a potential risk to patient safety and the quality of care. The data can therefore provide arguments for sufficient resource deployment for safely treating emergencies comprehensively.

The implementation of TNW aimed for improvement of communication and cooperation between trauma centers of different levels of trauma care. The survey addressed several items of pre- and post-transfer communication which was rated mostly good. This goal seems to be accomplished.

From the answers of the survey, it appears that transfer requests are not submitted in a standardized way. As mentioned before the main link between trauma centers is a non-standardized phone call. Within the regionalized networks trauma telephone numbers of the participating hospitals are published within the network and are quite well known. Submission of patient documents in a standardized way before transferring the patient could optimize the process. Some trauma centers take advantage of telecommunicative options that have been implemented within the TNW. TK-med^®^ offers the possibility to submit patient documents including x-rays and CT Scans in a safe and standardized way. The survey did not go in further details why TK-med^®^ had not been used more often when transferring patients. Availability as well as user friendliness could be possible reasons and should be addressed in future surveys. Standardized documentation of the patients demographics as well as the injury pattern and the actual vital status including radiographic findings could be easily transmitted via TK-med^®^ so that receiving hospitals already have original data before the patient arrives, in order to be better prepared and allocate necessary resources (19). Devecki et al. also showed a decrease in time before transfer after implementing a transfer protocol, one must consider the fact that these patients were transfer between Non-trauma Centers and Trauma centers (20).

The perception of direct feedback after transferring a patient has been rated differently by the transferring vs. receiving trauma centers. There seems to be space for future improvement. Especially the overall evaluation of the transferring process after the patient has been discharged only takes place in about 13%–18% of the cases. But this is one of the major fields for securing quality or potentially optimizing processes.

The aftercare of former severely injured patients seems to be a complex issue. The respondents stated that occasionally patients get sent back to the initial trauma center for further treatment or rehabilitation (10%–15%) but the majority of overall aftercare of the patients within an outpatient department takes place in Level-I Trauma Centers.

Thus resources need to be discussed within the TNW to provide aftercare concepts of severely injured patients.

The survey also addressed the question of a subjective overall judgement whether implementation of TNW has improved the management and the quality of care of severely injured patients or not. Most respondents judged that TNW had a somehow positive impact on the management of trauma patients. If we try to judge the effect of TNW on trauma patient care, overall measured by objective data (study I) and subjective perception (study II) good reasons could be announced, that TNW has improved the management and the quality of patient care significantly. This is especially true as TNW was initiated when more and more hospitals quit from trauma care for economical reasons.

## Conclusion

The implementation of TNW has improved the communication and quality of comprehensive trauma care of severely injured patients within Germany. By defining standards, working out guidelines and regulations, the primary allocation of patients has improved and so has the standard of care within the trauma centers of different levels of trauma care. If transfer is necessary, it is mostly organized and performed in an efficient matter. Although the transferring process seems to be working well, predictors such as higher level of head injury reveal that preclinical algorithm also present a potential of further improvement. So does the communication after a transfer has taken place to ensure and optimize high quality processes. A standardized protocol including a transfer-document should be implemented and existing resources should be used. The aftercare of severely injured patients needs to be focused on in future TNW-projects.

### Limitation

The main limitation of this study is the fact that the data of the transferred patients cannot be linked within the TR-DGU with those being received at the receiving trauma centers. Thus, the prehospital data is partly missing. The expected mortality rate (RISC-II-Score) cannot be calculated due to missing data of the transferring process.

The data collected in the German TR-DGU is not necessarily complete especially if the transfer takes place rapidly. Patients who died before a planned transfer are not included and some patients are transferred due to unknown, non-medical reasons.

Another limitation is the retrospective design of our study. The questionnaire has been sent out to the trauma centers, but only about 30% have handed in their replies. Interpretation of these subjective judgements and ratings needs to put into a sensitive and cautious surrounding to avoid false consequences.

## Data availability statement

The raw data supporting the conclusions of this article will be made available by the authors, without undue reservation.

## Author contributions

CS: Writing – original draft, Writing – review & editing, Conceptualization, Investigation. DB: Writing – review & editing, Data curation, Methodology. SR: Writing – review & editing. BB: Writing – review & editing. RH: Writing – review & editing. WL: Writing – review & editing. RL: Formal analysis, Writing – review & editing. HD: Conceptualization, Investigation, Writing – original draft, Writing – review & editing.
