# Supplementary material for: Evaluation of the interhospital patient transfer after implementation of a regionalized trauma care system (TraumaNetzwerk DGU®) in Germany
Source: Front Med (Lausanne). 2023 Nov 15;10:1298562. doi: 10.3389/fmed.2023.1298562 (PMC10684689; doi:10.3389/fmed.2023.1298562)
Supplement: Supplementary file 1 [file Table_1.DOCX]

Questionnaire

What is the structure of your Hospital?

1. Which Level does your Trauma Center belong to?

O Level I

O Level II

O Level III

1. For which level of occupational health procedure are you authorized?

O DAV (Level III)

O VAV (Level II)

O SAV (Level I)

1. Does your Trauma Center have an outpatient clinic for polytraumatized patients?

O Yes

O No

1. How many transferred patients did your clinic receive 2018 via trauma room?
2. How many of these patients did you receive from a hospital outside of your Regionalized Trauma Care System?
3. What are the most common reasons for transfers to you? Please name the top 4 reasons

O Higher Level of Trauma Care

O Not authorized for occupational health procedure due to work realted injury

O No ICU-capacity

O No OR-capacity

O Missing of special competencies (neurosurgery, heart surgery, ECMO etc.)

O Complications

O Economical reason

O Transfer to closest hospital to residence

O Rehabilitation

O other

1. After what time period did the transfer to your trauma center take place?

O less then 2 hours after the accident

O 2-6 hours after the accident

O 6-12 hours after the accident

O 12-24 hours after the accident

O 24-48 hours after the accident

O > 48 hours after the accident

1. How did the communication take place?

O Direct communication physician-physican

O Telefax

O Consultation

O other

1. How were documents and pictures mostly transmitted?

O Before the patient via fax, TKmed or other teleradiological connection

O With the patient by doctor’s letter and CD

O With the patient via doctor’s letter

O other

Do you give feedback to the transferring hospital?

1. Do you usually give feedback?

O Yes

O No

1. Do you usually give feedback about diagnosis and primary care?

O Yes

O No

1. Do you usually give feedback about quality of transfer (timing, communication, letter)

O Yes

O No

1. Do you usually send back a discharge letter?

O Yes

O No

1. Do you usually give other feedback?

O Yes

O No

Do you transfer the patients back after final care?

1. We transfer back rarely

O Yes

O No

1. Yes, after acute care/intensive care (even if there is still a need for surgery)

O Yes

O No

1. Yes, soon after complete care

O Yes

O No

1. Yes, if acute rehabilitation is already initialized

O Yes

O No

1. Yes, for outpatient after care

O Yes

O No

1. Do you see transferred patients in your outpatient clinic?

O Yes, often

O Yes, rarely

O No

1. Did you receive more transferred patients after initializing the TNW?

O Yes

O No

O Other

1. How many Patients did you transfer in 2018?
2. How many of them did you transfer outside of TNW?
3. How many patients did you transfer to specialized hospitals (children, burns, paraplegia)
4. What are the top 4 reasons for transfers in your trauma center?

O Higher Level of Trauma Care

O Not authorized for occupational health procedure due to work realted injury

O No ICU-capacity

O No OR-capacity

O Missing of special competencies (neurosurgery, heart surgery, ECMO etc.)

O Complications

O Economical reason

O Transfer to closest hospital to residence

O Rehabilitation

O other

1. After what time period did you transfer patients?

O less then 2 hours after the accident

O 2-6 hours after the accident

O 6-12 hours after the accident

O 12-24 hours after the accident

O 24-48 hours after the accident

O > 48 hours after the accident

1. How did the communication take place?

O Direct communication physician-physican

O Telefax

O Consultation

O other

1. How were documents and pictures mostly transmitted?

O Before the patient via fax, TKmed or other teleradiological connection

O With the patient by doctor’s letter and CD

O With the patient via doctor’s letter

O other

Do you get feedback from the hospitals you transfer to?

1. Do you usually get feedback?

O Yes

O No

1. Do you usually get feedback about diagnosis and primary care?

O Yes

O No

1. Do you usually get feedback about quality of transfer (timing, communication, letter)

O Yes

O No

1. Do you usually get a discharge letter?

O Yes

O No

1. Do you usually get other feedback?

O Yes

O No

Do you usually get those patients transferred back?

1. Rarely

O Yes

O No

1. Yes, after acute care/intensive care (even if there is still a need for surgery)

O Yes

O No

1. Yes, soon after complete care

O Yes

O No

1. Yes, if acute rehabilitation is already initialized

O Yes

O No

1. Yes, for outpatient after care

O Yes

O No

1. Do you see those patients in your outpatient clinic?

O Yes

O No

1. Did you transfer more transferred patients after initializing the TNW?

O Yes

O No

O Other

1. How does the TNW work from your perspective in behalf of transferring patients?

O Works good an has really improved the care of severly injured patients

O Sometimes it works good, sometime it does not, it improved the care of severly injured patients a bit

O Mostly it doesn’t work and didn’t bring any improvement

O other

Thank You for your engagement and taking the time
